# Paeonol enhances macrophage phagocytic function by modulating lipid metabolism through the P53-TREM2 axis

**DOI:** 10.3389/fphar.2023.1214756

**Published:** 2023-06-28

**Authors:** Jifei Miao, Xiaoming Liu, Yuanpin Liao, Yiwen Li, Yingyan Kuang, Juanxia Zheng, Zigang Li, Jiao Lan

**Affiliations:** ^1^ School of Chemical Biology and Biotechnology, Peking University Shenzhen Graduate School, Shenzhen, China; ^2^ Shenzhen Bao’an Traditional Chinese Medicine Hospital, Shenzhen, China; ^3^ Shenzhen Bao’an Traditional Chinese Medicine Hospital, Guangzhou University of Chinese Medicine, Guangzhou, China

**Keywords:** phagocytosis, lipid metabolism, TREM2, p53, macrophage

## Abstract

**Introduction:** The emerging concept of immunometabolism highlights the interplay between lipid metabolism and phagocytosis in macrophages. Triggering Receptor Expressed on Myeloid Cells 2 (TREM2) has been identified as an essential modulator of both lipid metabolism and phagocytic function in macrophages. This study aims to investigate the roles of P53 and TREM2 in regulating macrophage lipid metabolism and phagocytosis and to evaluate the potential therapeutic effects of paeonol on these processes.

**Methods:** CRISPR-Cas9 was utilized to generate P53 and TREM2 knockout RAW264.7 cell lines. The dual-luciferase reporter gene assay was performed to assess the interaction between P53 and the TREM2 promoter. A series of functional assays were conducted to evaluate the impact of P53 and TREM2 on macrophage lipid metabolism and phagocytic function. The effects of Paeonol on these processes were also examined.

**Results:** Our findings revealed that paeonol induces the accumulation of P53 in the nucleus. P53 acts as a transcription factor that upregulates the expression of TREM2, promoting macrophage lipid metabolism, metabolic activity, and phagocytic capacity. Additionally, dual-luciferase reporter gene assays confirmed the interaction between P53 and the TREM2 promoter.

**Discussion:** This study provides novel insights into the roles of P53 and TREM2 in regulating macrophage lipid metabolism and phagocytic function. Further research is warranted to explore the potential applications of Paeonol and to elucidate the molecular mechanisms underlying the observed effects.

## GRAPHICAL ABSTRACT

**Figure:**
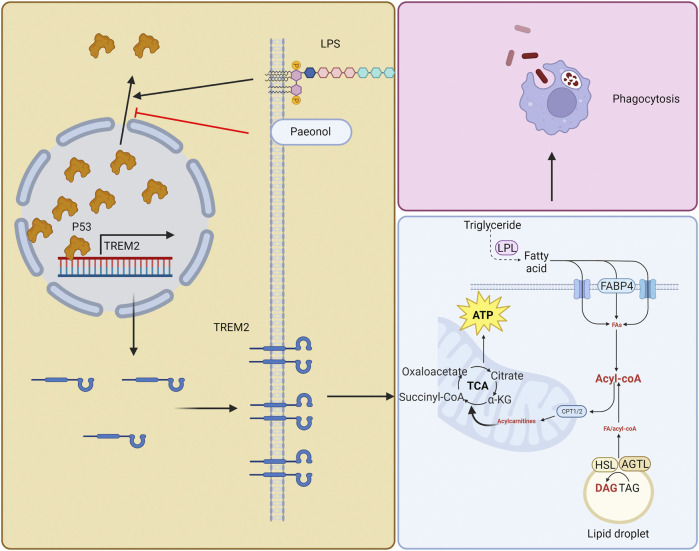
In this illustration, we depict a three-step process involving the roles of paeonol, TREM2, and ATP in macrophage function. Firstly, paeonol inhibits the export of P53 from the cell nucleus, leading to its accumulation within the nucleus. This, in turn, increases the likelihood of P53 binding to the TREM2 promoter, subsequently enhancing the expression of TREM2. Secondly, as TREM2 expression increases, it promotes lipid metabolism in macrophages. This enhancement of lipid metabolism subsequently leads to elevated levels of ATP. Lastly, with the increase in ATP levels, the phagocytic function of macrophages is enhanced, contributing to a more efficient immune response.

## Introduction

Macrophages are essential immune cells involved in host defense and tissue homeostasis, playing a pivotal role in the clearance of pathogens, apoptotic cells, and debris through the process of phagocytosis (Uribe-Querol and Rosales, 2020). The efficiency of macrophage phagocytic function is crucial in determining the outcome of inflammatory responses and host resistance to infections. Dysregulation of macrophage function can contribute to the pathogenesis of various inflammatory diseases and infections, such as atherosclerosis, obesity, and autoimmune disorders (Feng et al., 2021). Therefore, understanding the molecular mechanisms that regulate macrophage phagocytic function is of great importance for the development of novel therapeutic strategies to combat these diseases.

Lipid metabolism is an essential aspect of macrophage biology, as it influences cellular energy homeostasis, membrane composition, and signaling processes (Yan and Horng, 2020). Proper regulation of lipid metabolism, including the balance between anabolic and catabolic processes such as fatty acid synthesis and oxidation, is critical for maintaining macrophage function and overall immune system competence (Remmerie and Scott, 2018). In recent years, the link between lipid metabolism and phagocytosis has become increasingly evident, leading to the emerging concept of immunometabolism (O’Neill and Pearce, 2016). This interdisciplinary field aims to elucidate the intricate interplay between cellular metabolism and immune responses, providing insights into the development of novel therapeutic strategies.

In this context, Triggering Receptor Expressed on Myeloid Cells 2 (TREM2) has emerged as an important modulator of both lipid metabolism and phagocytic function in macrophages (Kleinberger et al., 2014). TREM2 promotes the uptake and catabolism of lipids, leading to enhanced macrophage metabolic activity, which in turn supports phagocytic capacity (Cantoni et al., 2015). Furthermore, the expression of TREM2 is increased by P53, a well-known tumor suppressor with pleiotropic functions in cellular processes, including metabolism (Tebaldi et al., 2015; Zajkowicz et al., 2018). The P53-TREM2 axis thus represents an important regulatory mechanism connecting lipid metabolism and macrophage phagocytic function.

Paeonol, a compound derived from Paeonia suffruticosa, has shown promise in enhancing macrophage phagocytic function in our preliminary studies (Miao et al., 2020). However, the impact of paeonol on macrophage lipid metabolism and the potential involvement of the P53-TREM2 axis remain to be elucidated. We found paeonol promotes the accumulation of P53 in the nucleus, so we guess paeonol could increase the expression of TREM2. In this study, we aimed to determine the effect of paeonol on macrophage lipid metabolism and investigate the role of the P53-TREM2 axis in modulating the phagocytic function of macrophages.

## Results

### Paeonol promotes the phagocytic function of macrophage

**FIGURE 1:**
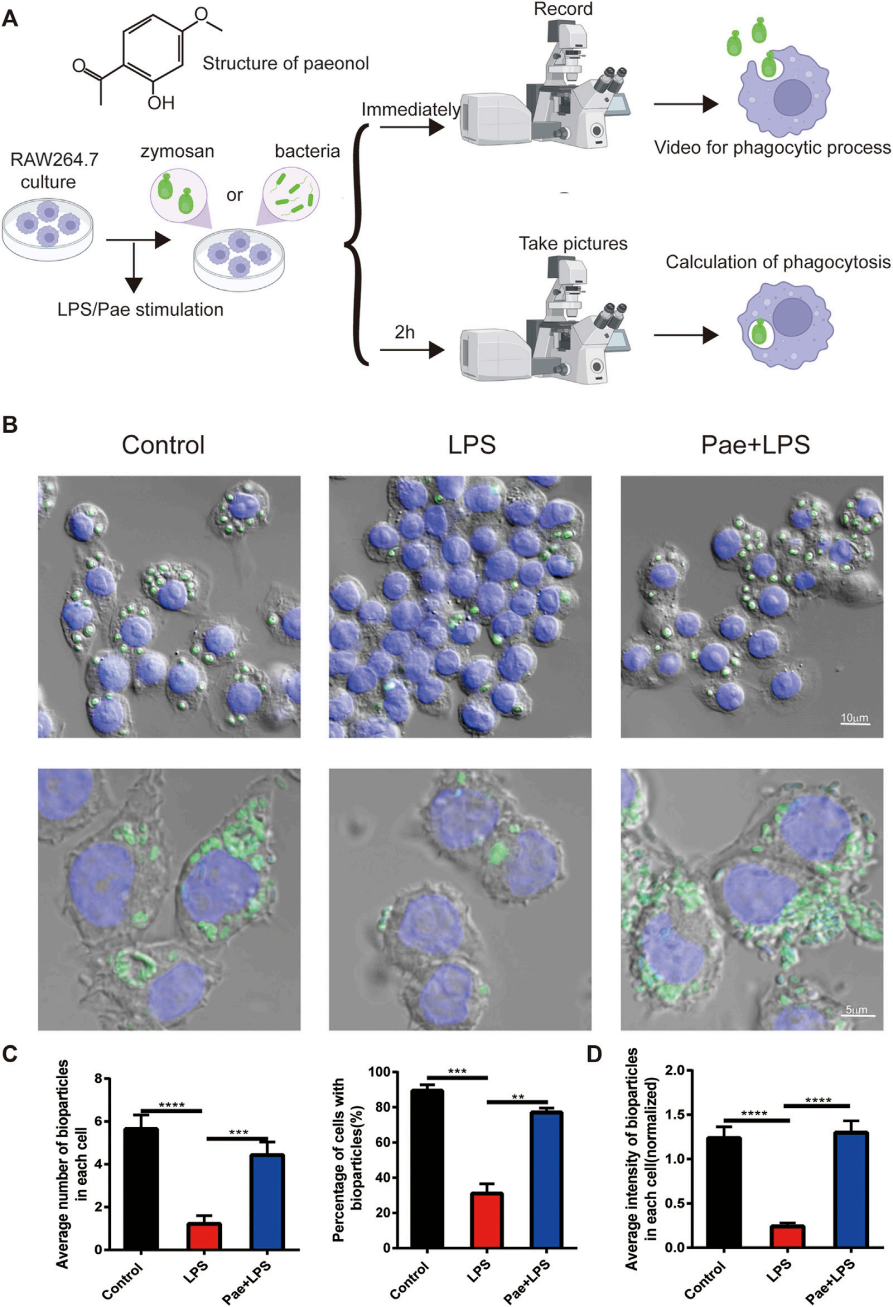
Paeonol promotes the phagocytic function of macrophage **(A)** The structure of paeonol and schematic illustration of the cell culture and phagocytic function assessment. **(B)** Microscopy images of macrophages engulfing particles, the upper layer green points corresponds to zymosan fluorescent bioparticles, the lower layer green points signifies bacteria fluorescent bioparticles, and the nuclei was stained with DAPI(blue). **(C)** A graphical summary of macrophage engulfment of yeast polysaccharide bioparticles, illustrating both the mean number of zymosan fluorescent bioparticles ingested per cell (left) and the percentage of cells containing zymosan fluorescent bioparticles (right). **(D)** A depiction of the fluorescence intensity data for macrophages engulfing bacteria fluorescent bioparticles. **p < 0.01, ***p < 0.001, ****p < 0.0001. Data represents the mean ± SEM of 3 independent experiments.

In our previous study, we found that continuous stimulation with lipopolysaccharide (LPS) for 3 days reduced the phagocytic function of macrophages, and paeonol promoted the phagocytic ability of macrophage (Miao et al., 2020). For better understanding of this study, we repeated the previous experiments. We used continuous LPS stimulation of RAW264.7 cells for 3 days as the model (LPS group), while the experimental group (Pae+LPS group) was subjected to 3 consecutive days of LPS+paeonol stimulation. Building on our prior research, we selected a concentration of 600 nM paeonol for this study. This choice was based on experiments in which we stimulated RAW264.7 cells with 200, 600, and 1,000 nM paeonol (Miao et al., 2021). We found that 600 nM paeonol effectively inhibited HMGB1 secretion, which induces P53 nuclear accumulation (which strongly co-localizes with HMGB1), without causing significant cellular damage. First, we co-cultured macrophages with zymosan fluorescent bioparticles and immediately recorded videos to observe the phagocytic process of macrophages (Figure 1A). We found that macrophages in control group were highly active, quickly extending pseudopodia towards zymosan fluorescent bioparticles and completing phagocytosis in a short time (Supplementary Video S1). In contrast, LPS-stimulated macrophages were relatively inactive, with only a few cells extending pseudopodia, and no migration towards or phagocytosis of bioparticles was observed (Supplementary Video S2). Macrophages in the Pae group, similar to the control group, quickly responded to surrounding bioparticles and completed phagocytosis (Supplementary Video S3).

Furthermore, we quantified the phagocytosis of bioparticles by macrophages after 2 h of co-culture. We found that macrophages in the control group engulfed an average of 5.64 bioparticles, 4.42 in the Pae group, and only 1.21 in the LPS group. Additionally, 89.3% of control group cells engulfed bioparticles, 77% in the Pae group, and only 31% in the LPS group (Figures 1B, C). To further investigate whether the differences in phagocytic function of macrophages in different groups were caused by specific cell surface receptors or other factors, we co-cultured macrophages with bacteria fluorescent bioparticles. We found that LPS and paeonol had similar effects on macrophage phagocytosis of bacteria as on zymosan fluorescent bioparticles (Figures 1B, D). These results are consistent with our previous findings that continuous LPS stimulation reduces macrophage phagocytic function, while paeonol rescues this phenomenon, and this process does not involve specific recognition receptors on the surface of macrophages.

### Paeonol promotes the lipid metabolism of macrophage

**FIGURE 2:**
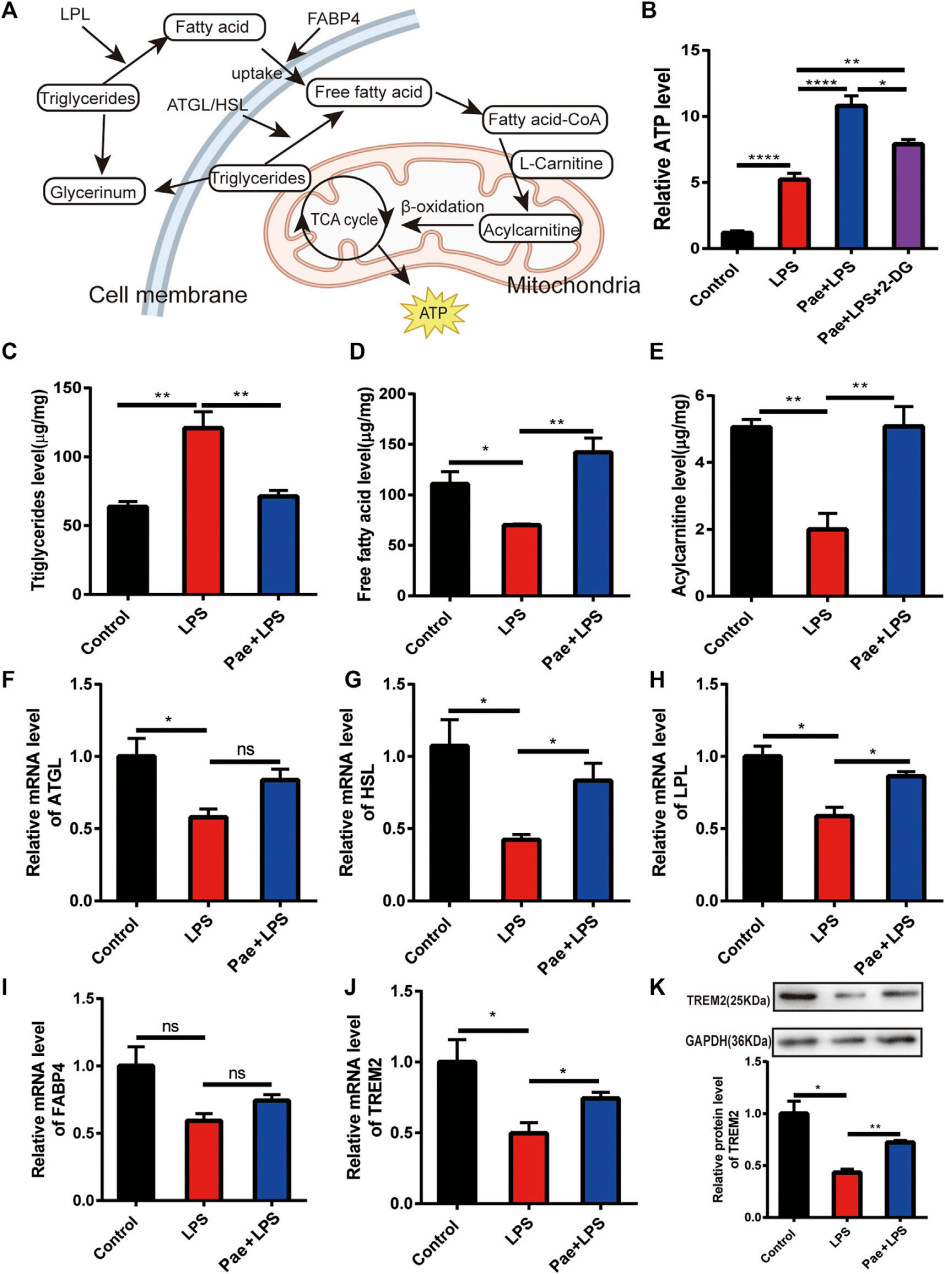
Paeonol promotes the lipid metabolism of macrophage **(A)** A simplified schematic diagram of macrophage lipid metabolism. **(B)** A bar graph illustrating ATP levels in different groups, normalized to the average value of the control group. **(C)** A bar graph showing the content of triglycerides in different groups. **(D)** A bar graph representing the content of free fatty acids in different groups. **(E)** A bar graph displaying the content of acylcarnitines in different groups. **(F)** A bar graph depicting the transcriptional levels of the ATGL gene in different groups. **(G)** A bar graph demonstrating the transcriptional levels of the HSL gene in different groups. **(H)** A bar graph presenting the transcriptional levels of the LPL gene in different groups. **(I)** A bar graph illustrating the transcriptional levels of the FABP4 gene in different groups. **(J)** A bar graph illustrating the transcriptional levels of the TREM2 gene in different groups. **(K)** A graphical representation and quantification of the expression levels of the TREM2 protein in different groups. *p < 0.05, **p < 0.01, ****p < 0.0001, ns *p* > 0.05. Data represent the mean ± SEM of 3 independent experiments.

LPS stimulation affects macrophage lipid metabolism, such as inhibiting fatty acid oxidation (Feingold et al., 2008) and suppressing the lipid metabolism (Guo et al., 2015), this maybe one reason why continuous LPS stimulation decrease the phagocytosis of macrophages. Therefore, to investigate the effect of paeonol on the lipid metabolism of microglial cells, we detected intermediate products of lipid metabolism (such as triglycerides, free fatty acids, and acylcarnitines) and some related proteins (such as LPL, ATGL, HSL, and FABP4) (Figure 2A). We first examined the ATP levels in macrophages and found that LPS stimulation increased ATP levels, which might be due to altered glucose metabolism in macrophages, with reduced oxidative phosphorylation and increased glycolysis promoting ATP production (Lavin et al., 2014). Paeonol stimulation further increased ATP levels, suggesting that paeonol might promote lipid metabolism and ATP production. We used 2-DG to inhibit glycolysis and found that ATP levels indeed decreased compared to the Pae+LPS group. However, compared to the LPS group, the Pae+LPS+2-DG group still showed a significant increase, with a statistically significant difference, which emphasized that glycolysis is not the main target of paeonol but lipid metabolism (Figure 2B).

We then examined the effect of paeonol on triglyceride, free fatty acid, and acylcarnitine levels in macrophages. We found that LPS stimulation significantly increased triglyceride levels compared to the control group, while free fatty acid and acylcarnitine levels significantly decreased. This suggests that LPS stimulation reduced the macrophage’s ability to break down triglycerides, leading to triglyceride accumulation, subsequently reducing downstream fatty acid activation and ATP production in mitochondria. Paeonol reversed this phenomenon by reducing the accumulation of triglycerides in cells and increasing the content of free fatty acids and acylcarnitines (Figures 2C–E). By detecting proteins, we found that paeonol increasing the expression of LPL, ATGL, and HSL, which are closely related to the breakdown of triglyceride (Figures 2F–H). Although there was no significant difference in FABP4 expression among the three groups, the trend suggested that paeonol might promote FABP4 expression (Figure 2I), possibly promoting lipid metabolism by increasing fatty acid transport.

In recent years, an increasing number of studies have shown that TREM2 is closely related to lipid metabolism (Jaitin et al., 2019; Li et al., 2022), and TREM2 affects macrophage phagocytosis and inflammatory responses by regulating lipid metabolism (Wolfe et al., 2018; Nugent et al., 2020). Therefore, we observed the effect of paeonol on TREM2 protein levels and found that LPS reduced TREM2 expression, while paeonol promoted TREM2 expression (Figures 2J, K). As a cell surface receptor, TREM2 is the starting point of lipid metabolism-related pathways, suggesting that paeonol’s regulation of lipid metabolism might be based on its control of TREM2.

### Paeonol promotes phagocytosis and lipid metabolism through TREM2

**FIGURE 3:**
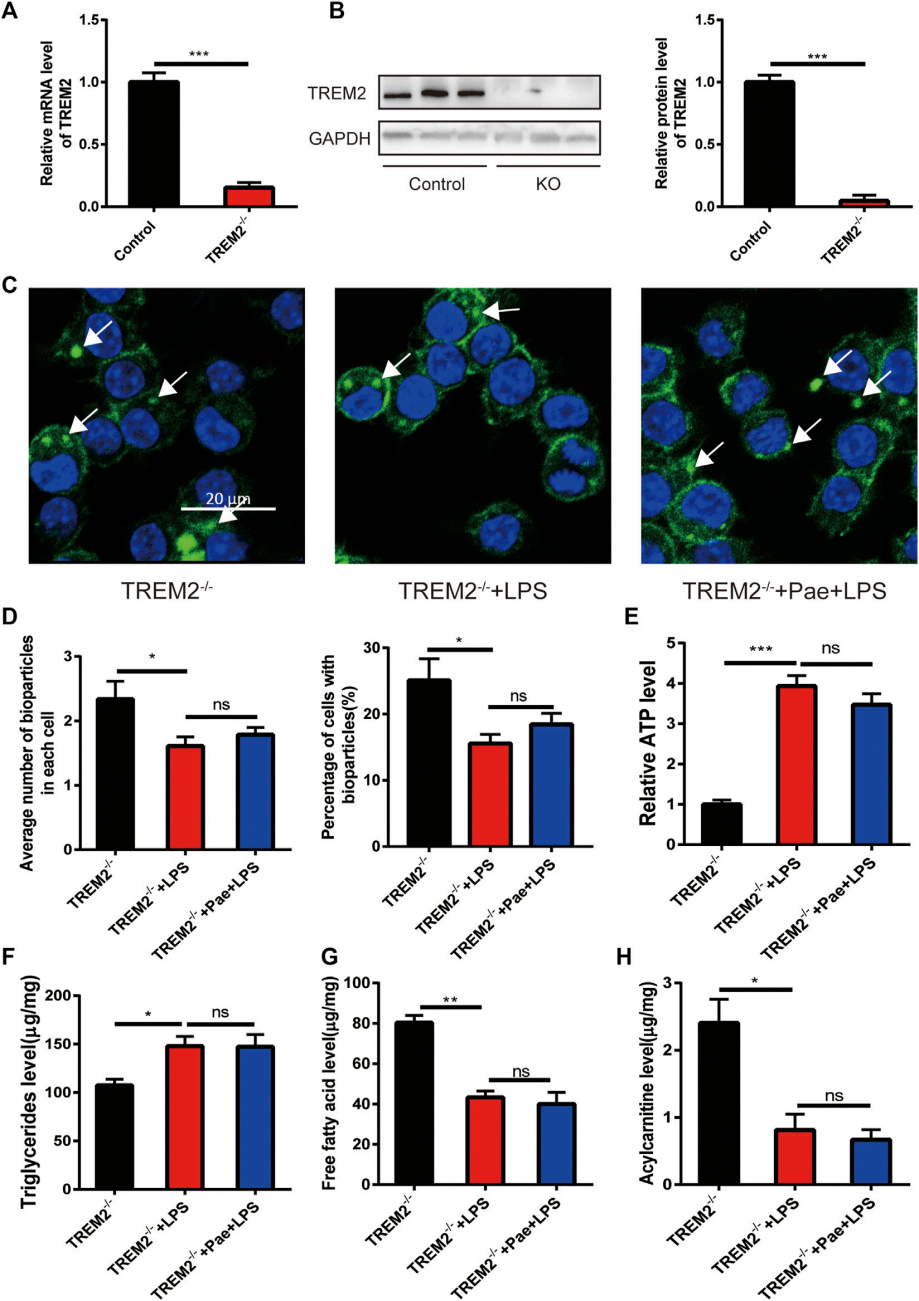
Paeonol promotes phagocytosis and lipid metabolism through TREM2 **(A)** RT-PCR analysis of TREM2 knockout efficiency. **(B)** Western blot analysis and quantification of TREM2 knockout efficiency. **(C)** Phagocytosis assay results in TREM2^-/-^ cells; white arrows indicate the ingested pHrodo™ Green Zymosan Bioparticles (green), and nuclei are stained with DAPI (blue). **(D)** A bar graph summarizing the phagocytosis results in TREM2^-/-^ cells, with the left bar chart representing the mean number of pHrodo™ Green Zymosan Bioparticles ingested per cell, and the right bar chart illustrating the percentage of cells containing pHrodo™ Green Zymosan Bioparticles. **(E)** A bar graph showing ATP levels in different groups, normalized to the average value of the control group. **(F)** A bar graph representing the content of triglycerides in different groups. **(G)** A bar graph depicting the content of free fatty acids in different groups. **(H)** A bar graph displaying the content of acylcarnitines in different groups. *p < 0.05, **p < 0.01, ***p < 0.001, ns *p* > 0.05. Data represent the mean ± SEM of 3 independent experiments.

To elucidate the specific role of TREM2 in the process of paeonol promoting macrophage phagocytosis and lipid metabolism, we constructed a TREM2 knockout macrophage cell line. Using qPCR and Western blot detection, we found that the cell line achieved a high TREM2 knockout efficiency (Figures 3A, B). We then applied the same LPS and paeonol stimulation as before to the TREM2 knockout cell line. We found that, compared to the previous phagocytosis function (Figure 1), knocking out TREM2 itself reduced macrophage phagocytosis, LPS stimulation further decreased phagocytosis in TREM2 knockout cells, and paeonol lost its ability to improve macrophage phagocytosis (Figures 3C, D). By observing the phagocytosis process, we found that after knocking out TREM2, the phagocytic ability of macrophages decreased, and we did not observe cell migration and phagocytosis towards bioparticles (Supplementary Video S4). After LPS(Supplementary Video S5) and paeonol (Supplementary Video S6) stimulation, the phagocytic activity of the cells was further reduced.

By further detecting lipid metabolism intermediates, we found that LPS also increased ATP levels and triglyceride accumulation in TREM2 knockout cells, and reduced free fatty acid and acylcarnitine levels, with paeonol not altering this phenomenon caused by LPS (Figures 3E–H). The above information indicates that the effect of paeonol on macrophage phagocytosis and lipid metabolism is mainly achieved by regulating TREM2.

### Paeonol promotes TREM2 expression by regulating P53 localization

**FIGURE 4:**
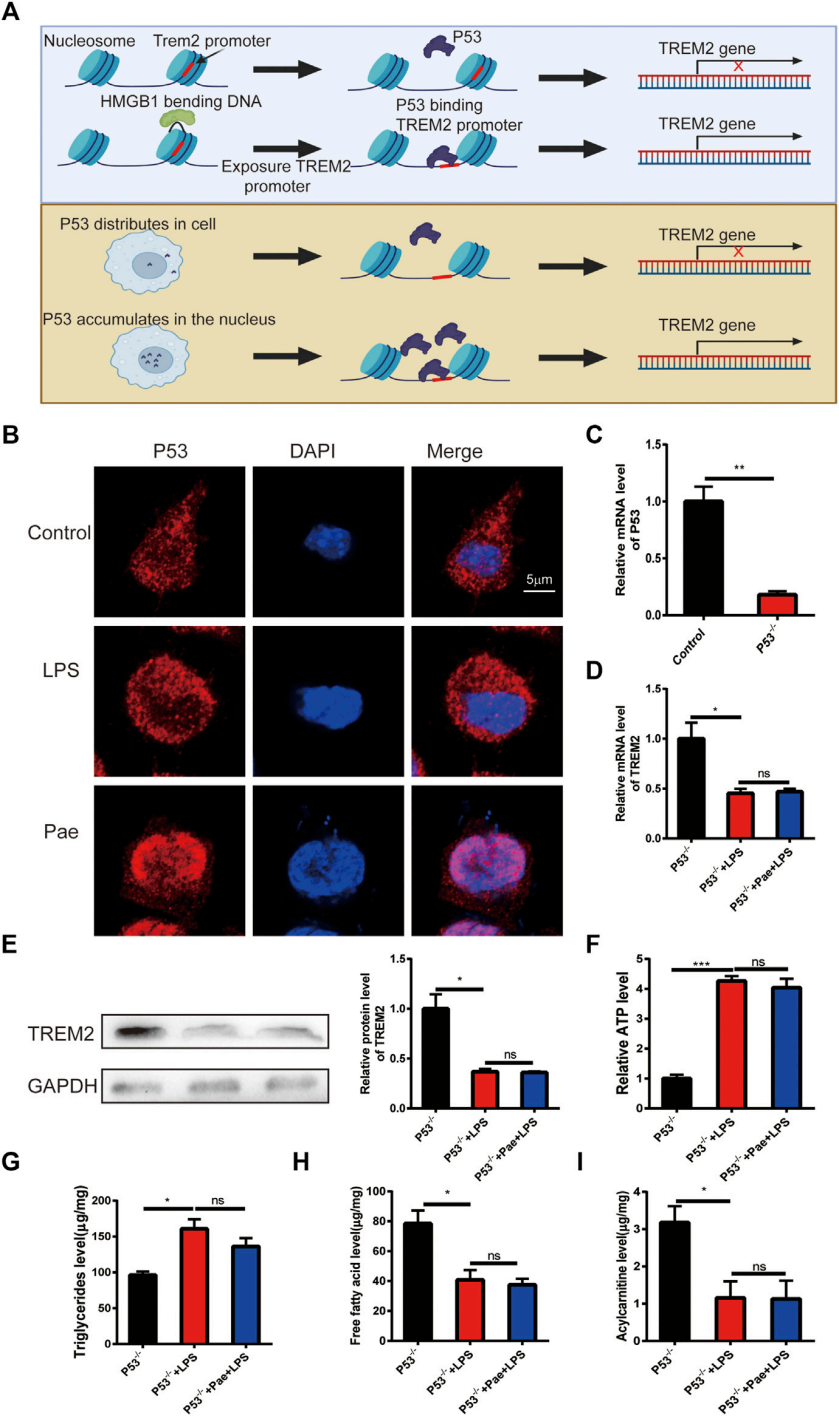
Paeonol promotes TREM2 expression by regulating P53 localization **(A)** Schematic illustration of two hypotheses: the upper panel suggests that HMGB1 might fold DNA to expose TREM2 promoter sites, increasing the probability of P53 binding; the lower panel proposes that increased nuclear P53 accumulation alone can enhance P53-mediated transcription of the TREM2 gene when the TREM2 promoter is exposed. **(B)** P53 localization results showing LPS promoting P53 localization in the cytoplasm and paeonol promoting P53 localization in the nucleus. **(C)** RT-PCR analysis of P53 knockout efficiency. **(D)** PCR-based quantification of TREM2 gene transcription levels in P53 knockout cells after LPS and paeonol stimulation. **(E)** Western blot analysis and quantification of TREM2 protein levels in P53 knockout cells after LPS and paeonol stimulation. **(F)** A bar graph showing ATP levels in different groups, normalized to the average value of the control group. **(G)** A bar graph representing the content of triglycerides in different groups. **(H)** A bar graph depicting the content of free fatty acids in different groups. **(I)** A bar graph displaying the content of acylcarnitines in different groups. *p < 0.05, **p < 0.01, ***p < 0.001, ns *p* > 0.05. Data represent the mean ± SEM of 3 independent experiments.

HMGB1 is a widely distributed nuclear protein similar to histones, and it has a strong intracellular localization with P53 (Livesey et al., 2012; Kang et al., 2014). In our previous study, we found that paeonol could inhibit the acetylation of HMGB1 and prevent its nuclear export, thereby inducing a large accumulation of P53 in the cell nucleus. Studies have shown that the promoter region of TREM2 contains cis-acting elements for P53, which can promote the transcription of the TREM2 gene. Therefore, we hypothesize that there might be two different molecular mechanisms to promote TREM2 transcription: 1) The promoter region of TREM2 is wrapped around the nucleosome, and HMGB1 can expose the promoter region of TREM2 by folding DNA, increasing the binding rate of P53 (Štros et al., 2018); 2) The promoter region of TREM2 is normally exposed, and simply increasing the concentration of P53 in the cell nucleus can increase the probability of P53 binding to the TREM2 promoter (Figure 4A). This study mainly focuses on the second hypothesis. We first observed the effects of LPS and paeonol on the intracellular localization of P53. We found that in normal macrophages, P53 is diffusely distributed throughout the cell. After LPS stimulation, P53 mainly concentrates in the cytoplasm, while after paeonol stimulation, P53 accumulates largely in the cell nucleus (Figure 4B).

To investigate the effect of P53 on TREM2 expression, we constructed a P53 knockout macrophage cell line (Figure 4C). By observing TREM2 expression, we found that LPS also reduced TREM2 expression, suggesting that LPS inhibits TREM2 expression through non-P53 pathways, and paeonol did not promote TREM2 expression, indicating that the effect of paeonol on TREM2 is mainly achieved through P53 (Figures 4D, E). We then detected lipid metabolism intermediates in P53 knockout cells and found that the effect of paeonol on these intermediates was consistent with that in TREM2 knockout cells (Figures 4F–I), indicating that the influence of paeonol on lipid metabolism is mainly achieved through the P53/TREM2 axis.

### P53 promotes the expression of TREM2

**FIGURE 5:**
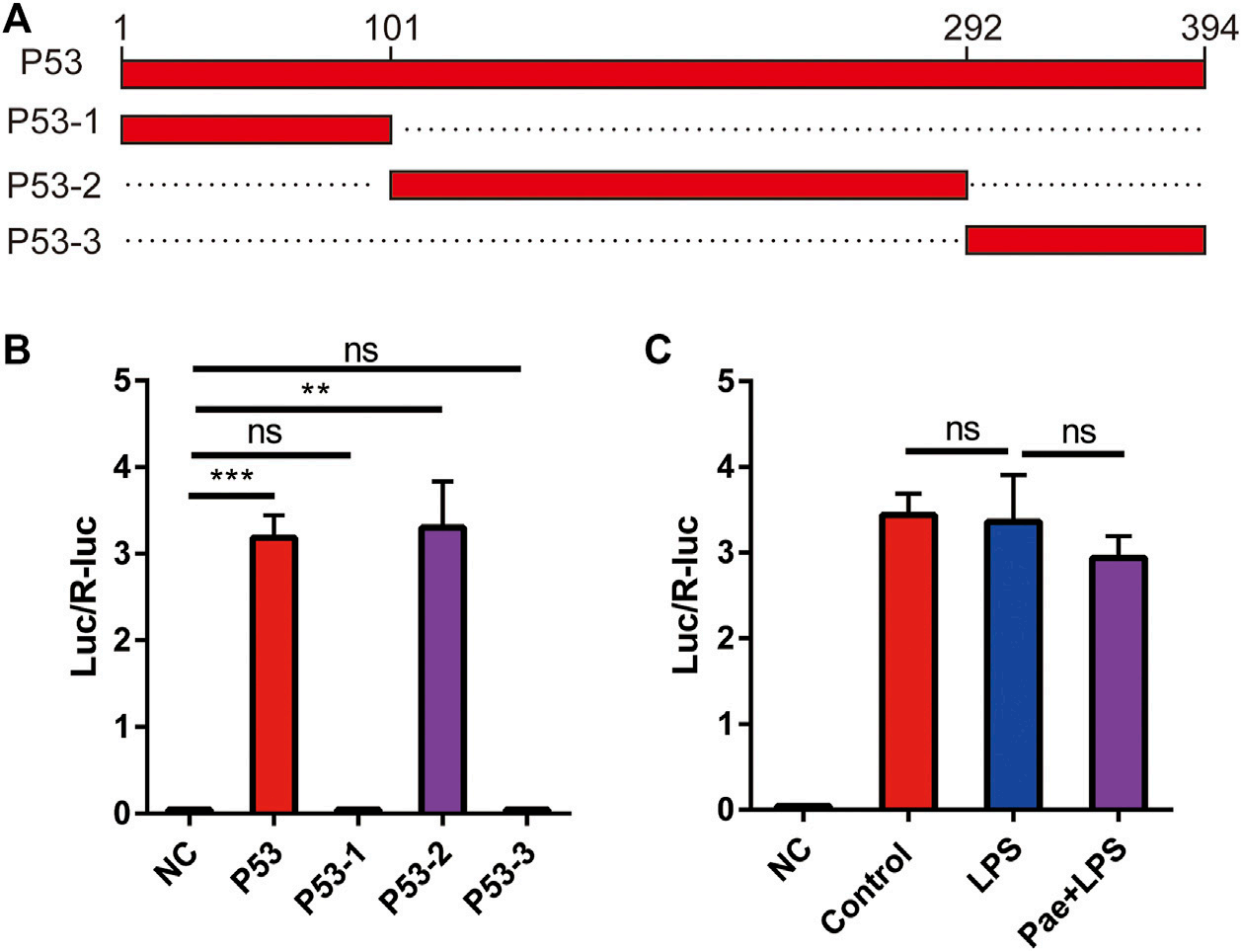
P53 promotes the expression of TREM2 **(A)** Schematic illustration of the four P53 truncation constructs. **(B)** A bar graph representing the results of the dual-luciferase reporter assay examining the effect of P53 truncations on TREM2 promoter activity, NC: negative control. **(C)** A bar graph displaying the impact of LPS and paeonol treatments on the ability of P53 to modulate TREM2 promoter activity, as assessed by the dual-luciferase reporter assay. **p < 0.01, ***p < 0.001, ns *p* > 0.05. Data represent the mean ± SEM of 3 independent experiments.

To further investigate the specific binding sites between P53 and the TREM2 promoter, we constructed four P53 expression plasmids, including: 1) P53: expressing full-length P53 protein; 2) P53-1: expressing P53 protein consisting of amino acids 1–101; 3) P53-2: expressing P53 protein consisting of amino acids 102–292; 4) P53-3: expressing P53 protein consisting of amino acids 293–394 (Figure 5A). By using dual luciferase reporter system, we co-transfected the above four plasmids with pGL4.10-TREM2 promoter plasmid into HEK293T cells to observe the activation effects of different truncated P53 proteins on the TREM2 promoter. We found that P53 and P53-2 proteins could promote the activation of the TREM2 promoter (Figure 5B), indicating that the area between amino acids 102–292 of P53 is the functional area. The specific site needs to be further investigated in subsequent experiments. In order to investigate whether LPS and paeonol influence the enhancing effect of P53 on TREM2 promoter expression, we performed experiments using HEK293T cells transfected with a luciferase-expressing TREM2 promoter plasmid. This experimental setup allowed us to specifically examine the modulation of TREM2 promoter activation by LPS and paeonol in the presence of P53, without considering the potential regulatory effects of P53 cellular localization. Our results demonstrated that neither LPS nor paeonol directly affected the enhancing role of P53 on TREM2 promoter activation (Figure 5C). These findings suggest that the effect of paeonol on TREM2 expression mainly depends on its regulatory role in P53 localization, rather than directly modulating the P53-mediated activation of the TREM2 promoter.

## Discussion

In this study, we aimed to elucidate the role of paeonol in modulating macrophage phagocytosis and lipid metabolism through the regulation of the P53/TREM2 axis. Macrophages play a critical role in immune defense and tissue homeostasis through phagocytosis, which is the process of engulfing and degrading pathogens, dead cells, and debris (Lancaster et al., 2019). Efficient phagocytosis is essential for maintaining immune system functionality and preventing inflammation-related diseases. Lipid metabolism is also crucial for macrophage function, as it provides energy and structural components necessary for macrophage activation, migration, and phagocytosis (Yan and Horng, 2020). Dysregulation of lipid metabolism in macrophages can lead to lipid accumulation, impaired phagocytosis, and chronic inflammation, contributing to the pathogenesis of various diseases, such as atherosclerosis, obesity, and diabetes (Remmerie and Scott, 2018).

Emerging evidence has demonstrated a connection between phagocytosis and lipid metabolism in macrophages, with TREM2 playing a vital role in linking these processes (Nugent et al., 2020). Our results demonstrated that paeonol treatment led to a significant increase in TREM2 expression and lipid metabolism, which in turn enhanced macrophage phagocytosis. The loss of TREM2 markedly reduced macrophage phagocytic capacity, highlighting the essential role of TREM2 in mediating the effects of paeonol on macrophage phagocytosis. Moreover, our data showed that the absence of TREM2 led to decreased macrophage migration towards bioparticles and reduced phagocytic activity upon LPS and paeonol stimulation. We also observed that paeonol promoted ATP production even when glycolysis was inhibited by 2-DG, confirming our hypothesis that paeonol acts through lipid metabolism to produce this phenotype. These findings suggest that paeonol might exert its effects on macrophage phagocytosis primarily through the regulation of TREM2 expression and lipid metabolism.

Although our study revealed the critical role of TREM2 in mediating the effects of paeonol on macrophage phagocytosis, further investigations are needed to explore the exact molecular mechanisms underlying TREM2-mediated phagocytosis enhancement, such as the involvement of other signaling pathways or effector molecules. Our study also uncovered a novel mechanism by which paeonol modulates TREM2 expression through the regulation of P53 localization. We found that paeonol treatment led to the accumulation of P53 in the nucleus, which in turn facilitated P53 binding to the TREM2 promoter and enhanced TREM2 expression. Additionally, we observed that LPS stimulation caused P53 to concentrate in the cytoplasm, which might be associated with reduced TREM2 expression. The importance of the P53/TREM2 axis in the effects of paeonol was further supported by our findings that loss of either P53 or TREM2 abolished the ability of paeonol to enhance macrophage phagocytosis and lipid metabolism. Furthermore, we demonstrated that the binding site for P53 on the TREM2 promoter lies within the region of amino acids 102–292. Further studies are required to identify the specific binding site and elucidate the precise molecular mechanisms underlying P53/TREM2 interaction.

### Limitations and future directions

Although our study provided novel insights into the role of paeonol in enhancing macrophage phagocytosis and lipid metabolism through the P53/TREM2 axis, several limitations should be addressed. First, our study relied solely on *in vitro* experiments, which might not fully reflect the *in vivo* physiological conditions. Future research should involve animal models of macrophage-associated diseases to validate our findings and assess the therapeutic potential of paeonol. Second, our study focused on the effects of paeonol on macrophage function, but other immune cells, such as neutrophils and dendritic cells, might also be affected by paeonol treatment. Investigating the impact of paeonol on other immune cell types could further elucidate the immunomodulatory effects of paeonol and provide a more comprehensive understanding of its therapeutic potential.

## Conclusion

In conclusion, our study demonstrated that paeonol enhances macrophage phagocytosis and lipid metabolism through the regulation of the P53/TREM2 axis. These findings not only advanced our understanding of the molecular mechanisms underlying the effects of paeonol on macrophages but also highlighted the potential of paeonol as a therapeutic agent for the treatment of diseases associated with macrophage dysfunction. Future studies should investigate the *in vivo* effects of paeonol in animal models of macrophage-associated diseases, explore the impact of paeonol on other immune cell types, and further elucidate the molecular mechanisms underlying the P53/TREM2 interaction.

## Materials and method

### Cell culture and treatment

The RAW 264.7 cell line (Shanghai Institute of Cell Biology, Shanghai, China) was cultured in Dulbecco’s Modified Eagle’s Medium (DMEM) supplemented with 10% fetal bovine serum (FBS; Gibco; Thermo Fisher Scientific, Inc., Waltham, MA, United States) and antibiotics (100 U/mL penicillin and 100 mg/mL streptomycin) under a humidified atmosphere containing 5% CO_2_ at 37°C. Upon reaching 70%–80% confluence, RAW264.7 cells were transferred to serum-free medium. To investigate the effects of LPS on the phagocytic capabilities of RAW264.7 cells, cells were exposed to LPS (0.2 μg/mL) for 3d. In the Pae+LPS group, cells were treated with LPS (0.2 μg/mL) and paeonol (600 nM) for 3d.

### Phagocytosis video shooting

RAW264.7 cells were placed in glass petri dishes (diameter = 20 mm) at a density of 5 × 10^4 cells/dish and subjected to the previously described culture and treatment conditions. After the cells were settled, they were placed in a live-cell imaging chamber of a laser scanning confocal microscope, which was maintained at a constant 5% CO2 atmosphere. Subsequently, zymosan fluorescent bioparticles (2 μL) or bacteria fluorescent bioparticles (2 μL) (Thermo Fisher Scientific) were added to the cell culture dish. Immediately, continuous imaging of the same field of cells was initiated, capturing images every 5 s for a total duration of 30 min. Afterward, all captured images were combined into a single video output for further analysis.

### Phagocytic function analysis

Round glass coverslips were placed in 6-well plates, and RAW264.7 cells were seeded on the coverslips at a density of 1 × 10^6 cells/well. The cells were cultured and treated according to the previously described conditions. Afterward, the cells were co-cultured with zymosan fluorescent bioparticles (2 μL) for 2 h. Subsequently, the cells were washed three times with room temperature phosphate-buffered saline (PBS) for 10 min each. The cells were then fixed with 4% paraformaldehyde (PFA) for 10 min, followed by another three washes with PBS. The cell nuclei were stained with DAPI for 10 min, and the cells were washed again three times with PBS. The coverslips were carefully removed from the plate, with the cells facing inward, and mounted onto glass slides with mounting medium. Finally, images were immediately captured using a laser scanning confocal microscope. The average quantity of bioparticles per cell and the proportion of cells containing bioparticles were determined in a single-blind manner.

### ATP assay

ATP levels in each group were assessed utilizing ATP Assay Kits (Beyotime, S0026) following the manufacturer’s guidelines. After rinsing the cells twice with PBS, 200 μL of lysis buffer from the ATP detection kit was added to each tube, followed by ultrasonication. The lysates were then centrifuged at 12,000 g for 5 min at 4°C. Supernatants were collected into fresh 1.5 mL tubes for ATP analysis using the ATP detection kit. Relative ATP levels were determined using the formula: relative ATP level = ATP value/protein value. The protein content of the samples was measured at 562 nm using a Multiscan MK3 instrument from Bio-Rad. ATP levels in the control group were normalized to a value of 1.

### Triglyceride, free fatty acid and acylcarnitine levels assay

The levels of triglycerides, free fatty acids, and acylcarnitines in RAW264.7 cells were determined utilizing triglyceride assay (Abcam, ab65336), free fatty acid assay (Abcam, ab65341), and acylcarnitine assay (Shanghai mIBio Biotechnology Company, XK-SJH-A437), respectively. These assays were performed in accordance with the manufacturer’s protocols.

### RT-PCR

Total RNA was extracted from RAW264.7 cells using Trizol reagent, following the manufacturer’s guidelines (Invitrogen). The reverse transcription process was carried out using ReverTra Ace qPCR RT Master Mix (Toyobo, FSQ-201). RNA concentrations were adjusted to 2 gl^−1^ in nuclease-free water, and a 20 µL reaction volume was employed for reverse transcription of total RNA. Real-time quantitative RT-PCR was performed using SYBR Green (Roche) reagent to amplify cDNA. Sequence of primers are shown in Table 1. Triplicate samples were assessed, and GAPDH served as an internal control. Data from qRT-PCR were obtained using a Roche LightCycler 480II real-time PCR machine and corresponding software.

**TABLE 1:** Sequence of primers used for RT-PCR.

Gene	Forward:5′-3′	Reverse:5′-3′
ATGL	ACG​CAC​ATC​TAC​GGC​GCC​TCG​G	GCT​CAT​GGC​TAT​CAG​CAG​GCA​GGA​C
HSL	GAG​GCG​ACC​TAT​GGA​ACC​TTG​T	ATC​CAT​TGC​CCG​GCG​TCT​GCT​C
LPL	GCT​ACC​TGT​CAT​TTC​AAT​CAC​A	CCA​CCA​GTT​TGG​TGT​AGC​CCG​C
FABP4	GTG​ATG​CTT​TTG​TAG​GTA​CCT​GG	CTG​GCC​CAG​TAT​GAA​GGA​AAT​C
TREM2	AGA​AGG​GCC​CAT​GCC​AGC​GTG​T	TTC​CTG​AGG​GTG​TCA​GCC​TCA​C
P53	GAC​CCA​GGT​CCA​GAT​GAA​GCT​C	GAG​TAC​GTG​CAA​GTC​ACA​GAC​A

### Western blot

RAW264.7 cells were homogenized in ice-cold lysis buffer (RIPA) for 40 min and then centrifuged at 12,000 rpm for 10 min at 4 °C. Supernatants were collected in a fresh 1.5 mL tube, and protein samples were subjected to sodium dodecyl sulfate-polyacrylamide gel electrophoresis (SDS-PAGE) before being immunoblotted onto polyvinylidene difluoride (PVDF) membranes. Samples containing 30 µg of protein were separated using 10% SDS-PAGE gels. Proteins were transferred to PVDF membranes in an ice-cold buffer (25 mM TrisHCl, 192 mM glycine, and 20% methanol) via electrotransfer for 1.5 h. Immunoblots were probed with the primary antibodies (TREM2, rabbit, 1:1,000, ab305103; GAPDH, mouse, 1:2000, ab8245). Goat-anti-mouse and goat-anti-rabbit secondary antibodies were obtained from Millipore (AP132P, AP124P, 1:3,000). Band intensities were quantified, normalized to GAPDH, and averaged across at least three independent experiments.

### Immunofluorescence

Round glass coverslips were placed in 6-well plates, and RAW264.7 cells were seeded on the coverslips at a density of 1 × 10^6 cells/well. The cells were cultured and treated according to the previously described conditions. Subsequently, the cells were washed three times with room temperature phosphate-buffered saline (PBS) for 10 min each. The cells were then fixed with 4% paraformaldehyde (PFA) for 10 min, followed by another three washes with PBS. The cells were permeabilized and blocked using PBS containing 0.5% Triton X-100% and 10% normal goat serum at room temperature for 1 h. Then, cells were incubated with anti-P53 antibody (mouse, 1:1,000, ab26) in blocking buffer at 4°C overnight. Following PBS washing, secondary antibody (goat anti-mouse 555, 1:500, ab150118) were added to the blocking buffer and allowed to incubate for 1 hour. After washing, the cell nuclei were stained with DAPI for 10 min, and the cells were washed again three times with PBS. The coverslips were carefully removed from the plate, with the cells facing inward, and mounted onto glass slides with mounting medium. Finally, images were immediately captured using a laser scanning confocal microscope.

### P53/TREM2 knockout RAW264.7 cells construction

Guide RNA (gRNA) sequences targeting the exon regions of the mouse p53 or TREM2 genes was designed by using chopchop tool. P53:5′-ATTTCACCCTCAAGATCCGCGGG-3’; TREM2:5′-GAGGAGTCATCGAGTTTCGAGGG-3’. The designed gRNA sequences will be cloned into a lentiviral CRISPR/Cas9 vector, lentiCRISPR v2, allowing for co-expression of Cas9 nuclease and gRNA. Recombinant lentiviruses can then be produced by co-transfecting the lentiCRISPR vector along with packaging plasmids into HEK293T cells. After harvesting and concentrating the viral supernatant, RAW264.7 cells can be transduced with the lentiviral particles containing the p53 or TREM2-specific gRNA and Cas9. Following transduction, successfully modified cells can be selected using Ampicillin. Lastly, knockout efficiency was validated by performing RT-PCR or assessing protein expression levels through Western blot analysis.

### Dual-luciferase reporter assay

Four p53 truncation expression plasmids were generated, including: 1) P53: expressing full-length P53 protein; 2) P53-1: expressing P53 protein consisting of amino acids 1–101; 3) P53-2: expressing P53 protein consisting of amino acids 102–292; 4) P53-3: expressing P53 protein consisting of amino acids 293–394. The TREM2 promoter region was cloned into the pGL4.10 vector containing the firefly luciferase reporter gene. Subsequently, HEK293T cells were co-transfected with each of the four p53 truncation expression plasmids and the TREM2-pGL4.10 construct. An internal control, the Renilla luciferase reporter gene driven by CMV promoter, can be co-transfected for normalization purposes. Following 24 h incubation to allow for expression of the transfected genes, cells will be harvested, and the dual-luciferase reporter assay was performed according to the manufacturer’s instructions. The firefly luciferase activity was normalized to Renilla luciferase activity to account for variations in transfection efficiency. The activation of the TREM2 promoter by each p53 truncation construct was compared to determine the potential regulatory effect of p53 on TREM2 expression.

### Data and statistical analysis

All results in the figures and text are expressed as the means ± SEM. The data were evaluated using GraphPad Prism Version 7.0 (GraphPad Software, La Jolla, CA, United States). The difference between two groups was analyzed using Student’s unpaired *t*-test. A value of *p* < 0.05 was considered significant for all tests.

## Data Availability

The original contributions presented in the study are included in the article/Supplementary Material, further inquiries can be directed to the corresponding author.
